# Supplementary material for: Chemical Constituent Profiling of Paecilomyces cicadae Liquid Fermentation for Astragli Radix
Source: Molecules. 2019 Aug 14;24(16):2948. doi: 10.3390/molecules24162948 (PMC6721272; doi:10.3390/molecules24162948)
Supplement: Supplementary file 1 [file molecules-24-02948-s001.pdf]

Supplementary Data Captions

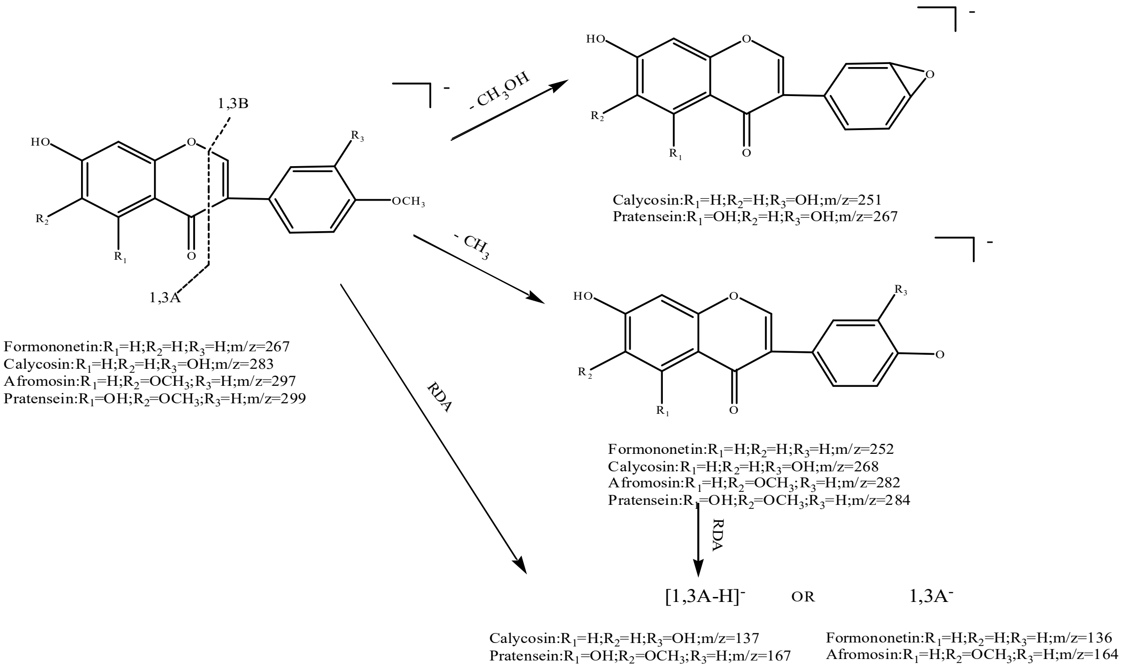

Figure. S1 Proposed fragmentation pathways for representative flavonoids detected in negative ion mode

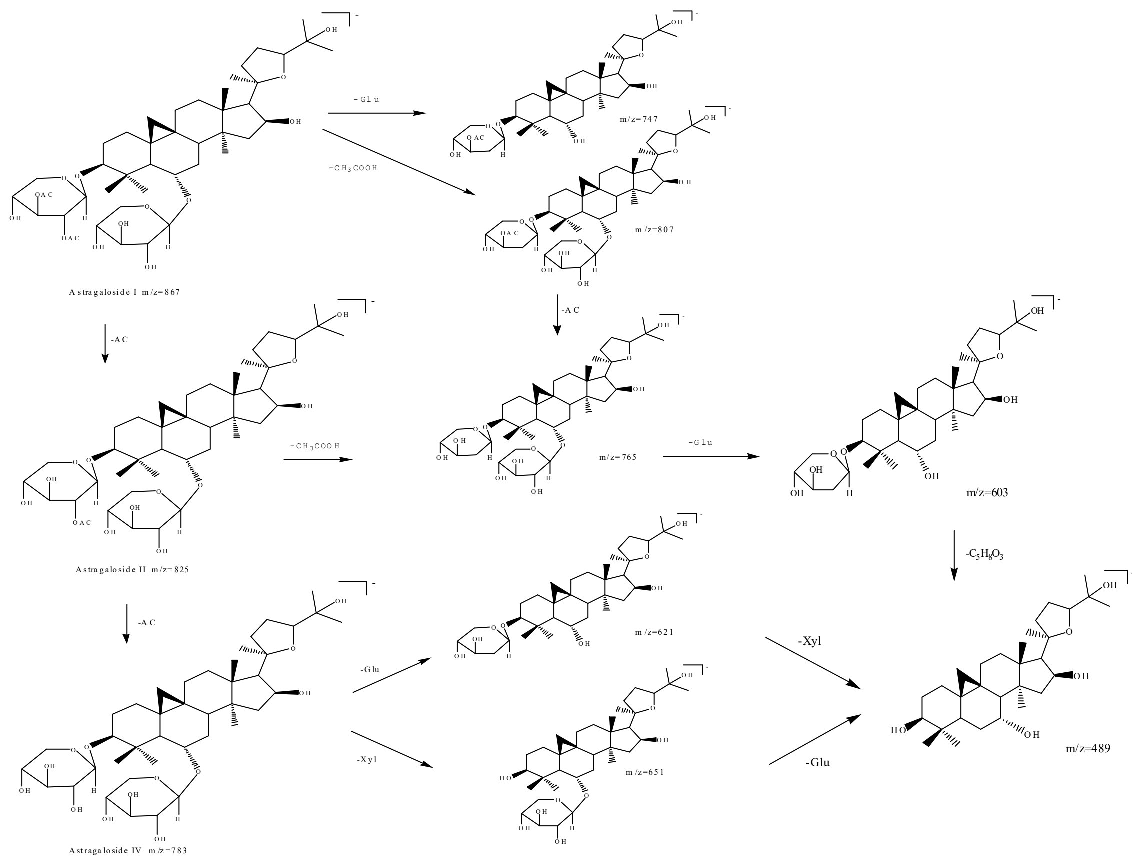

Figure. S2 Proposed fragmentation pathways for representative saponin detected in negative ion mode
